# Comparison of the Androgenic Response of Spring and Winter Wheat (*Triticum aestivum* L.)

**DOI:** 10.3390/plants9010049

**Published:** 2019-12-31

**Authors:** Dorota Weigt, Angelika Kiel, Idzi Siatkowski, Joanna Zyprych-Walczak, Agnieszka Tomkowiak, Michał Kwiatek

**Affiliations:** 1 Department of Genetics and Plant Breeding, Poznań University of Life Sciences, 11 Dojazd St, 60-637 Poznań, Poland; dorota.weigt@up.poznan.pl (D.W.); agnieszka.tomkowiak@up.poznan.pl (A.T.); michal.kwiatek@up.poznan.pl (M.K.); 2 Department of Mathematical and Statistical Method, Poznań University of Life Sciences, 28 Wojska Polskiego St, 60-637 Poznań, Poland; idzi.siatkowski@up.poznan.pl (I.S.); joanna.zyprych@up.poznan.pl (J.Z.-W.)

**Keywords:** androgenesis, anther culture, doubled haploid, spring wheat, winter wheat, induction medium

## Abstract

Androgenesis is potentially the most effective technique for doubled haploid production of wheat. It is not however widely used in breeding programmes due to its main limitation: the genotype dependence. Due to genetic differences between spring and winter wheat, it was assumed that both phenotypes are different in their capacity to conduct androgenesis. And so, the aim of this investigation was to verify the effectiveness of androgenesis induction and plant regeneration of spring and winter wheat genotypes while considering varying amounts of growth hormones in the induction medium. Fifteen genotypes of spring wheat and fifteen of winter wheat were used in the experiment. Six hundred anthers of each of the 30 genotypes were plated and analysed. Previous studies have allowed selection of the best medium for wheat androgenesis and a combination of growth hormones that are the most effective in stimulating microspore proliferation. Therefore, C17 induction media with two combinations of growth hormones were used: I—supplemented only by auxins (2,4-D and dicamba), and II—supplemented by auxin and cytokinin (2,4-D and kinetin). Data was recorded according to the efficiency of androgenic structure formation (ASF), green plant regeneration (GPR), and albino plant regeneration (APR). The results showed that the induction and regeneration of androgenesis in the spring wheat were more efficient than in the winter ones. The spring genotypes formed more androgenic structures and green plants on anthers plated on the medium supplemented only by auxins, in contrast to the winter genotypes which were better induced and regenerated on the medium supplemented by auxin and cytokinin. The study showed that to increase the efficiency of androgenesis, it is necessary to select appropriate factors such as concentration and type of hormones in medium composition, affecting the course of the culturing procedure according to the winter or spring phenotype of donor plants.

## 1. Introduction

The potential of androgenesis in doubled haploid plant production is high because one anther contains over a thousand microspores and a new plant may develop from each of them [1]. The process takes place under artificial laboratory conditions, where immature male spores develop into haploid plants or spontaneously doubled haploids (DH). Microspore cells are formed as a result of the meiotic division, which causes their genetic diversification [2]. Apart from that, a spontaneous or induced doubling of the haploid genome results in full homozygosity of regenerated plants [3]. For this reason, haploids are particularly desirable for genetic, molecular and biochemical studies, as well as for breeding [4,5].

Application of DH lines allows breeders to introduce new genetic variations to plant material in a shorter time than is required with conventional methods [4]. For this reason, in some countries, cultivars generated with DH lines have an essential position in the seed market [6]. For example, in Canada, DH cultivars account for more than a third of all wheat sown. Lillian and AC Andrew are the most common cultivars in this country. Five years after the Glossa cultivar was obtained by androgenesis and released into the market, it was grown in 16% of the total area of wheat fields in Romania [6].

In spite of numerous studies on wheat androgenesis, there is still low induction of this process and regeneration of green plants. The emergence of albino plants and low frequency of the doubled number of chromosomes in haploid plants are significant problems [7,8]. It is difficult to develop an efficient method of regeneration of plants in anther cultures because there are a lot of factors influencing the efficiency of androgenetic induction [9]. The success in the regeneration of doubled haploids mostly depends on the genotype of donor plants [5]. It is also determined by their environment, the type and duration of exposure to stress, the culturing conditions—especially the kind of induction medium—the content of hormones in it and the interaction between these factors [10,11]. Due to the strong genotype dependence, many researchers suggest that input materials for androgenesis should be crossbred with well-regenerating cultivars [9]. As this is not always possible, an alternative solution is to adjust the methodology to winter and spring wheat genotypes separately. The first step is to analyse differences between them in reaction to the hormone content in the medium affecting microspores during androgenesis.

Spring and winter wheat cultivars essentially differ in their sensitivity to low temperatures and the photoperiod. Apical meristems of young winter seedlings must be exposed to low temperatures for a minimum period of time to trigger recessive vernalisation alleles and initiate flowering. Apart from that, in the process of evolution, winter cereals developed a wide range of traits, e.g., tolerance to low temperatures, which enhance their survival under difficult conditions [3]. Spring and winter phenotypes differ genetically and physiologically [12]. Therefore, as can be expected, their efficiency of regeneration in anther cultures will differ according to the external stimuli used in the experiment, especially low temperatures to which spikes are exposed during a thermal shock. When a stress factor is applied, microspores are reprogrammed from gametophytic into sporophytic development [2]. This transformation results in the development of haploid plants and there are often spontaneously doubled haploids [3]. Due to differences in tolerance to stress conditions of spring and winter wheat, we can expect that applying low temperature to spikes will cause stronger induction of microspores in spring or rather in winter genotypes.

Mononucleate microspores in anthers are also affected by substances contained in the culture medium. The hormonal composition of the induction medium is considered to be particularly significant [2]. According to reports in reference publications, it should mainly contain auxins [13]. However, some researchers claim that the addition of cytokinin increases the effectiveness of regeneration [6,10,14]. Due to physiological differences between spring and winter wheat, they may exhibit different reactions to hormonal stimulation. It is vital to select adequate hormone composition for both to increase the efficiency of mitotic divisions of microspores in anther cultures [2]. Consequently, we were interested in the influence of applied combinations of hormones in the induction medium on the capacity to conduct androgenesis of the studied genotypes.

There were few studies on wheat androgenesis which compared the efficiency of regeneration of plants from anthers of spring and winter wheat. However, there was a small number of genotypes described in these studies, so it was difficult to provide a clear answer as to whether spring or winter genotypes are better regenerated in anther cultures [4,10]. It is necessary to analyse androgenic efficiency in a larger number of genotypes of different origin. Therefore, this study aimed to compare the efficiency of androgenesis induction and plant regeneration of large number of spring and winter wheat genotypes with diverse genetic backgrounds.

## 2. Results

The efficiency of androgenesis in wheat anther cultures was investigated through observation of 600 anthers isolated from each of the 30 cultivars (15 spring and 15 winter). In total, 18,000 anthers were analysed.

### 2.1. Induction of Androgenesis

Androgenesis was induced in the anthers of all the genotypes under analysis. It was manifested by the proliferation of microspore cells and formation of androgenic structures. The efficiency of this process depended both on the genotype of donor plants and on the type of growth hormones added to the induction medium.

**Figure 1:**
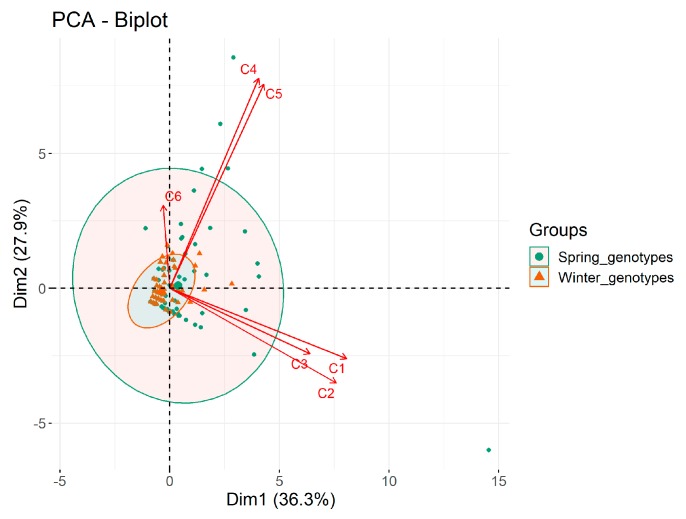
A PCA biplot showing the influence of the media on regeneration. C1, C2, C3—the androgenic structures, green plants and albino plants from the spring genotypes, respectively. C4, C5, C6—the androgenic structures, green plants and albino plants from the winter genotypes, respectively.

The anthers from the spring genotypes produced almost two times more AS than the anthers from the winter genotypes. The ASF amounted to 6.3 in the spring wheat and 3.2 in the winter ones (Table 1). In both groups there was a significant dependence between the ASF and the genotype of donor plants regenerated on medium I. On average, the ASF value of the spring wheat genotypes in both media (I and II) ranged from 2.0 (Rescue, Fortuna) to 18.3 (Tioga), whereas in the winter genotypes it ranged from 0.3 (KS96WGRC36) to 11.2 (Hondia). As results from the PCA in Figure 1 showed, the genotype influenced the androgenic response in the spring wheat to a greater extent than in the winter ones, as evidenced by the 95% confidence ellipsoids for both groups.

**Figure 2:**
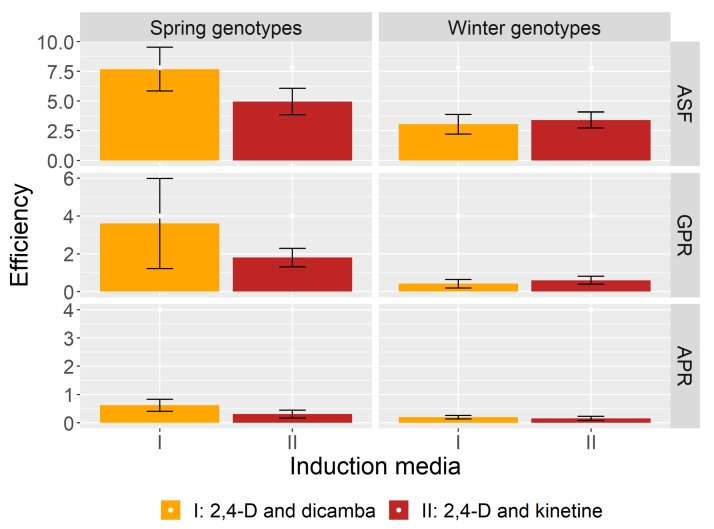
The efficiency of androgenic structures formation (ASF), green plants regeneration (GPR) and albino plants regeneration (APR) of the spring and winter genotypes according to the type of growth hormones in the induction media (I—containing auxins only and II—containing auxin and cytokinin); mean value ± standard error of mean (SEM).

It is noteworthy that ASF from microspores of the spring genotypes was greater on medium I, supplemented only with auxins (on average 7.7 androgenic structures per 100 plated anthers), than on medium II, containing auxin and cytokinin (on average 4.9 per 100). As far as the winter varieties are concerned, the induction of androgenesis was slightly higher on medium II, which contained kinetin (Figure 2). In regards to results from the right-sided Wilcoxon test (Table 2), where the ASF of spring and winter genotypes was compared according to the type of growth hormones in the induction medium, there were statistically significant differences observed in ASF but only on medium I (*p*-value < 0.05).

### 2.2. Regeneration of Green Plants

Green plants were obtained from 23 out of the 30 wheat genotypes under study (Table 1). There was no regeneration observed in one spring genotype and in six winter ones. The PCA shows that the influence of the genotype on plant regeneration was much stronger in the spring wheat (Figure 1). There was considerable diversification in the total number of plants formed: 243 from the spring genotypes and 46 from the winter ones. The highest GPR on both media was 20.0 in the spring variety AC Abbey and 2.7 in the winter cultivar Hondia (Table 1). The mean GPR in all the spring genotypes was five times greater than in the winter (2.7 and 0.5, respectively). Apart from that, more than 42% of AS developed green plants from the spring varieties, whereas regeneration was observed in 15.3% of AS from the winter genotypes.

The GPR was influenced by the composition of the induction medium as well. The dependence on the content of hormones was similar to the dependence observed in the ASF. The GPR in the spring wheat was higher in medium I (supplemented only with auxins), whereas in the winter varieties it was higher in medium II, containing auxin and cytokinin (Figure 2). The above results are confirmed by the Wilcoxon test where the dependence between differences in the GPR and the composition of induction medium proved to be significant in the spring and winter genotypes (*p*-value < 0.005), as can be seen in Table 2. There were considerable differences in the GPR on media I and II between most of the genotypes. Some genotypes regenerated only on one type of substrate (Table 1). The AC Abbey cultivar, characterised by the highest GPR, regenerated much more efficiently on medium I, producing 36.7 plants per 100 anthers in comparison to the 3.3 plants per 100 anthers on medium II.

### 2.3. Regeneration of Albino Plants

The regeneration of albino plants (AP) was also observed in the experiment. In total 42 albino plants developed from the anthers of spring wheat and 16 from the winter ones. The winter genotypes tended to form fewer albinos and they exhibited lesser regeneration of green plants. Only 8 out of 15 winter genotypes produced plants with chlorophyll defects, whereas 11 spring genotypes regenerated albinos (Table 1). The appearance of albino plants was also related to the type of the induction medium. There were slightly more albinos formed on medium I regardless of the wheat varieties under analysis (Figure 2). The highest APR amounted to 2.7 in the Leda Collection A47 spring genotype and 1.0 in the winter cultivar Clark. The differences were not significant in the influence of the induction medium on APR in the spring and winter plants (Table 2).

## 3. Material and Methods

### 3.1. Plant Material

Fifteen spring wheat and fifteen winter wheat genotypes were the research material. The plant material came from gene banks: National Small Grain Collection, United States Department of Agriculture, Agricultural Research Service Aberdeen-Idaho (USA) and Leibniz—Institut für Pflanzengenetik und Kulturpflanzenforschung—Gatersleben (Germany), from Polish breeding companies: DANKO Plant Breeding Ltd. Plant Breeding Smolice Ltd. IHAR Group, Malopolska Plant Breeding Ltd. and from Agriculture and Agri-Food Canada (AAFC), Semiarid Prairie Agriculture Research Centre, Swift Current (Canada). The plant material used in the experiment had a diverse pedigree, as shown in Table 3.

The donor plants were grown in 2015 and 2016 in an experimental field of the Department of Genetics and Plant Breeding, Poznań University of Life Sciences. The winter genotypes were sown in mid-October in the year preceding the experiment (2015). The spring varieties were sown in early April 2016. The weather conditions in years 2015 and 2016 are presented in Tables S1 and S2. Anther donor plants were fertilized with Azofoska (1 N:0.5 P_2_O_5_:1.4 K_2_O) twice: before germination (3 kg per 100 m^2^) and three weeks after germination (3 kg per 100 m^2^). There were no phytosanitary sprays applied.

### 3.2. Initial Treatment of Donor Plants

**Figure 3:**
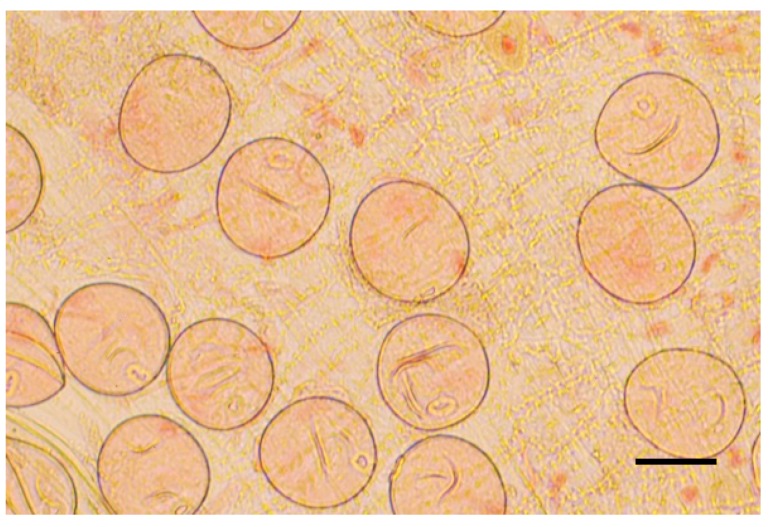
Microspores at the mononuclear stage suitable for anther culture. Bar represents 50 μm.

The spikes for anther isolation were harvested when their microspores were at the mid- or late mononuclear stage (Figure 3). The developmental stage of microspores was determined using preparations made from crushed anthers collected from the central part of the spike, which were stained with acetocarmine [15]. Tillers with spikes at the proper stage were stored in Erlenmeyer flasks with tap water in the dark at 4 °C for seven days.

### 3.3. Preparation and Course of In Vitro Cultures

After exposure to low temperatures, the spikes were superficially sterilised in a 4.85% sodium hypochlorite solution (Sigma-Aldrich^®^, St. Louis, MO, USA) for 4 min in a laminar flow cabinet. Next, the spikes were rinsed three times in sterile distilled water for 5 min. Then anthers were isolated from the spikes and placed on 50-mm Petri dishes (NOEX, Komorniki, Poland) containing a C17 induction medium in an amount of 8 mL per 1 Petri dish [16] modified according to Weigt et al. [17]. The modification was as follows: maltose replaced sucrose as a source of carbon; the sugar concentration was increased from 30 g L^−1^ to 90 g L^−1^; the medium was solidified using 2.5 g L^−1^ Gelrite^®^ (Sigma-Aldrich^®^). The induction medium was supplemented with growth hormones in two combinations: I—containing auxins only—with 1 mg L^−1^ of 2,4-dichlorophenoxyacetic acid (2,4-D) + 1 mg L^−1^ of dicamba, and II—containing auxin and cytokinin—with 1.5 mg L^−1^ of 2,4-D + 0.5 mg L^−1^ of kinetin (Sigma-Aldrich^®^). The medium was autoclaved for 27 min at a temperature of 121 °C and pressure of 121.6 kPa. Three hundred anthers of each genotype were placed on both I and II induction media—50 anthers from the central part of the spike per Petri plate. The plates with the anthers were tightly sealed with Parafilm M^®^ (Sigma-Aldrich^®^) and stored in darkness at a temperature of 28 °C for 6–8 weeks. After that period, all the plates were systematically observed and newly formed androgenic structures (AS) were counted and passaged to the MS regeneration medium [18], which was prepared according to the modifications developed by Weigt et al. [19], where 0.5 mg L^−1^ of NAA and kinetin was added. The medium was solidified with agar (Sigma-Aldrich^®^) concentrated at 0.6% (*w*/*v*) and it was autoclaved as described above. The plates with AS were sealed with Parafilm M^®^ and stored in the culture room at a temperature of 24 °C and a 16/8-h (light/dark) photoperiod. The plants’ regeneration was observed after 2–4 weeks (Figures S1 and S2). The regenerated green and albino plants (GP and AP, respectively) were counted. The GP were transferred to a fresh MS regeneration medium in glass flasks and they were tightly sealed with aluminium foil. After three weeks, the plants were replanted into pots with soil.

### 3.4. Data Analysis

To present the efficiency of androgenesis, the following parameters were calculated: androgenic structure formation (ASF)—the number of androgenic structures per 100 anthers, which also indicated the efficiency of androgenesis induction; green plant regeneration (GPR)—expressed as the number of green plants per 100 anthers; albino plant regeneration (APR)—the number of albino plants per 100 anthers. The above parameters are presented in Table 1 and Figure 2 (data supported by statistical calculations included in supplemental materials—see Table S4).

At the first stage of statistical analyses, the Shapiro-Wilk test did not reveal a normal data distribution. Therefore, the non-parametric right-sided Wilcoxon test was used for further comparisons of ASF, GPR and APR of the tested varieties according to the type of growth hormones in the induction media (I—2,4-D and dicamba, II—2,4-D and kinetin) (Table 2). Looking at Table 1, we can suppose that the spring genotypes were better regenerated on medium I, whereas the winter genotypes were better regenerated on medium II. Therefore, the hypotheses comparing the influence of medium I and medium II were verified with the right-sided Wilcoxon test for the spring wheat and the left-sided Wilcoxon test for the winter genotypes, but no statistically significant differences were found (Table S3).

Principal Component Analysis (PCA) was also used to assess the influence of different growth hormones in the induction medium and the ASF, GPR and APR of spring and winter wheat and also the relationship between observed variables. The result of PCA was presented as a biplot (Figure 1).

The R software, version 3.4.0 [20], was used for statistical calculations.

## 4. Discussion

The genotype of donor plants strongly determines haploid production. There are differences in the androgenic response observed between different species but also within the same species, where some genotypes effectively regenerate while others do not at all [6]. Reference publications provide contradictory data concerning androgenesis of spring and winter wheat [6,10,12,21]. Therefore, this study was an attempt to compare the androgenesis of the large number of spring and winter genotypes in wheat. The diverse genetic backgrounds of investigated plants and large number of plated anthers ensured reliable results. The ASF from the spring genotypes was twice as high as from the winter ones. The GPR from the spring wheat genotypes was even four times greater. There were different results in the study conducted by Zamani et al. [21]. They proved that the induction of embryo-like structures (ELS) was more efficient in winter wheat. However, their experiment showed that the GPR from the spring genotypes was much higher than from the winter cultivar. This finding was in agreement with our observations. Chaudhary et al. [10] studied the androgenetic capacity of nine elite winter wheat genotypes and two spring wheat genotypes. They observed that the spring genotypes were characterised by a higher efficiency of callus formation and green plant regeneration. The GPR ranged from 0 to 83.9 in the spring varieties and from 0 to 72.8 in the winter (calculated as number of green plant per number of calli). Grauda et al. [11] analysed the androgenetic capacity of 16 winter hybrids and five spring ones. They observed that the embryogenesis in the winter genotypes was worse than in the spring ones. The winter wheat hybrids were characterised by higher GPR.

Differences in the efficiency of androgenesis between spring and winter varieties were also researched in other cereals. Studies on triticale anther cultures mainly describe winter genotypes [22,23,24]. Ślusarkiewicz-Jarzina and Ponitka [25] investigated the androgenetic capacity of 34 winter triticale hybrids and 31 spring hybrids, and they found that the winter genotypes regenerated more androgenic structures and green plants per 100 anthers than the spring ones. However, Ślusarkiewicz-Jarzina et al. [26] conducted another experiment on 5 spring and 5 winter triticale F_1_ hybrids. They observed that the spring genotypes produced more androgenic structures and green plants per 100 anthers than the winter ones. However, the result was caused by supplementation of the medium with colchicine. This finding points to the fact that spring and winter varieties differ in their reaction to exogenous factors and that it is necessary to adjust the methodology individually to them. Makowska et al. [3] attempted to compare the androgenetic capacity of spring and winter barley F1 hybrids. They studied 20 genotypes and showed that the winter barley anthers regenerated androgenic structures and green plants more efficiently.

The hormonal composition of the medium is a factor determining the results of experiments. The presence of an exogenous auxin (2,4-D, picloram and dicamba are the most common auxins) is considered to be a sine qua non for regeneration to take place. Zheng and Konzak [27] observed callus induction without auxin, but the number of these structures was small, and they were incapable of regeneration to become plants. They also found that the optimal concentration of 2,4-D in the induction medium was 1–2 mg L^−1^. Gorbunova et al. [28] obtained calluses from anthers placed on a medium with the minimum 2,4-D concentration of 0.5 mg L^−1^. Auxin are often used together with cytokinin, which stimulate the mitotic division of cells (kinetin, BAP and zeatin are the most common cytokinins) [10,14,29]. The comparison of the reaction of the spring and winter wheat undergoing androgenesis to the composition of hormones in the medium showed differentiation in their efficiency of formation of calluses and green plants. Statistical analysis revealed that when the induction medium was supplemented only with auxins (medium I supplemented with 2,4-D and dicamba), the efficiency of regeneration of spring wheat increased significantly, as compared to the medium supplemented with 2,4-D and kinetin (medium II). Supplementation with cytokinin stimulated microspores of the winter varieties and increased the number of calluses and green plants in these genotypes. However, this tendency was not constant because the reaction of some of the spring and winter genotypes under analysis to the hormonal composition of the medium differed from the mean values. The GP regeneration of winter plant was, on average, more efficient in genotypes whose anthers had been placed on a medium supplemented with kinetin (medium II). Such a beneficial effect of auxins on spring wheat genotypes and cytokinin on winter ones was also observed in our experiment investigating the androgenic ability of F_1_ wheat hybrids (data not published). Zamani et al. [21] also observed better regeneration of winter genotypes on an induction medium supplemented with kinetin. Chaudhary et al. [10] added kinetin to stimulate the division of wheat microspore cells and observed that spring wheat regenerated more efficiently than winter. Ślusarkiewicz-Jarzina et al. [25] described triticale anther cultures. They supplemented the induction medium with 2,4-D and kinetin and they observed that winter genotypes produced haploids more efficiently. Makowska et al. [3] used the N6L induction medium with 0.5 mgL^−1^ of NAA, 0.5 mgL^−1^ of kinetin and 2.0 mgL^−1^ of 2,4-D. They observed that winter triticale exhibited a higher capacity to form plants. These results were not unequivocal, but the efficiency of androgenesis tended to increase in winter genotypes on media with cytokinin. This situation may have been caused by differences in the hormonal metabolism of spring and winter wheat resulting from the thermal shock applied in these experiments. It is known that endogenous phytohormones are released when plants are exposed to stress. The amount and type of these hormones determine the demand of cells for exogenous hormones provided in the substrate [30]. Additionally, it is necessary to remember that winter wheat is more tolerant of low temperatures. This fact might cause differences in the level of endogenous hormones released during cold stress in cells of winter and spring genotypes and thus have influence on the efficiency of androgenesis.

In our studies, the addition of growth hormones to the induction medium also affected the number of plants with chlorophyll defects. The research proved that, on average, there were more albino plants observed in the medium supplemented by 2,4-D and dicamba compared to 2,4-D and kinetin in winter as well as spring genotypes. However, this finding was not confirmed in our earlier studies on spring genotypes with higher resistance to *Fusarium* [17] and wheat leaf rust [19]. Regeneration of albino plants is genetically conditioned [3,21]. The cause of this phenomenon in plants obtained by androgenesis is the inability for proplastids to transform into chloroplasts [31]. Albinism occurs in androgenesis-derived plants in majority of cereals (wheat, barley, rye, triticale, rice and oat). The frequency of albino regenerants in cereals may vary from 5–100% of regenerated plants. Within the same species there are often observed genotypes in which albinism is more frequent than in others [31,32]. This thesis was confirmed in the study conducted by Weigt et al. [33]. The authors compared the efficiency of androgenesis of solid, medium and hollow-stemmed wheat genotypes, and they observed that solid-stemmed cultivars formed more albino plants on the medium containing 2,4-D and kinetin, whereas hollow-stemmed cultivars formed more albino plants on the medium with 2,4-D and dicamba.

## 5. Summary

The difference in the androgenic response between spring and winter wheat indicates a better capacity to support anther culture from the first group. There were only two winter genotypes versus 11 spring ones with GPR of 1% or more. Moreover, our results suggest that there is a possibility to increase the efficiency of androgenesis in both wheat phenotypes with the use of appropriate hormones affecting anther cultures. Supplementation of the induction medium with auxins only (2,4-D and dicamba) stimulates the androgenesis more effectively in spring genotypes. In winter genotypes however, the ASF and GPR increased when they were placed on a medium containing both auxin and cytokinin (2,4-D and kinetin). In addition, it was found that among the analysed spring genotypes, the AC Abbey was the most androgenic cultivar. Of the winter genotypes, the most effective embryogenesis and GPR were observed from anthers of the Hondia variety.

## Figures and Tables

**Table 1:** The average efficiency calculated for both tested media combinations of androgenic structures formation (ASF), green plants regeneration (GPR) and albino plants regeneration (APR) from the spring and winter wheat genotypes.

Phenotype	Genotype	ASF			GPR			APR		
		I	II	Avg.	I	II	Avg.	I	II	Avg.
Spring	Tybalt	2.7	1.7	2.2	2.3	0.0	1.2	0.0	0.3	0.2
	Rescue	0.7	3.3	2.0	0.0	2.7	1.3	0.7	0.0	0.3
	Fortuna	4.0	0.0	2.0	1.3	0.0	0.7	0.7	2.0	1.3
	Leda Collection A47	5.3	0.7	3.0	1.3	0.7	1.0	2.7	0.0	1.3
	Ostka Smolicka	8.0	1.3	4.7	0.0	1.3	0.7	0.0	0.0	0.0
	Ruzynska II	4.0	8.0	6.0	2.7	6.7	4.7	0.0	0.0	0.0
	Carola	4.7	3.3	4.0	0.0	2.7	1.3	1.3	0.7	1.0
	AC Abbey	24.7	6.0	15.3	36.7	3.3	20.0	2.0	0.0	1.0
	Tioga	23.3	13.3	18.3	1.3	3.3	2.3	0.7	0.0	0.3
	Parabola	3.3	4.7	4.0	0.0	0.0	0.0	0.0	0.7	0.3
	Chinook	5.3	11.3	8.3	0.0	3.3	1.7	0.0	0.7	0.3
	Glenman	10.7	10.0	10.3	0.7	0.0	0.3	0.0	0.0	0.0
	Arabella	5.0	1.3	3.2	0.7	1.7	1.2	1.0	0.3	0.7
	Sawtana	10.0	8.7	9.3	4.0	1.3	2.7	0.0	0.0	0.0
	Sumai 3	3.7	0.7	2.2	3.0	0.0	1.5	0.3	0.0	0.2
	**Mean**	7.7	4.9	6.3	3.6	1.8	2.7	0.6	0.3	0.5
Winter	Greer	2.3	1.7	2.0	0.0	0.0	0.0	0.0	0.0	0.0
	KS96WGRC36	0.7	0.0	0.3	0.3	0.0	0.2	0.0	0.0	0.0
	Wichita	3.0	5.0	4.0	0.0	0.3	0.2	0.0	0.0	0.0
	Geneva	1.3	0.7	1.0	0.0	0.0	0.0	0.3	0.3	0.3
	Freedom	1.3	2.7	2.0	0.0	0.3	0.2	0.0	0.0	0.0
	Lr19	1.3	1.3	1.3	0.0	0.0	0.0	0.0	0.0	0.0
	Augusta	0.3	0.7	0.5	0.0	0.0	0.0	0.0	0.0	0.0
	Century	1.7	2.3	2.0	0.0	0.0	0.0	0.0	0.0	0.0
	Antelope	6.7	5.7	6.2	0.3	0.3	0.3	0.7	0.3	0.5
	Agrus	3.7	2.3	3.0	0.0	0.0	0.0	0.3	0.0	0.2
	Clark	1.0	3.7	2.3	0.3	1.0	0.7	0.3	1.0	0.7
	Ozon	6.0	5.3	5.7	2.0	2.0	2.0	0.0	0.3	0.2
	Hondia	12.3	10.0	11.2	3.0	2.3	2.7	0.7	0.0	0.3
	Karl 92	0.7	4.3	2.5	0.0	1.3	0.7	0.3	0.0	0.2
	Tam 107	3.3	5.3	4.3	0.3	1.3	0.8	0.3	0.3	0.3
	**Mean**	3.0	3.4	3.2	0.4	0.6	0.5	0.2	0.1	0.2

I—induction medium containing 2,4-D + dicamba; II—induction medium containing 2,4-D + kinetin.

**Table 2:** A comparison of the efficiency of androgenic structures formation (ASF), green plants regeneration (GPR) and albino plants regeneration (APR) of the spring and winter varieties according to the type of growth hormones in the induction media based on right-sided Wilcoxon test.

**I Induction Medium (2.4-D and Dicamba)—The Right-Sided Wilcoxon Test**		
**Parameters**	**The Value of Wilcoxon’s Statistics**	***p*-Value**
ASF (no of androgenic structures/100 plated anthers)	177.0	0.004 **
GPR (no of green plants regeneration/100 plated anthers)	160.0	0.020 *
APR (no of albino plants regeneration/100 plated anthers)	140.5	0.109
**II Induction Medium (2.4-D and Kinetine)—The Right-Sided Wilcoxon Test**		
**Parameters**	**The Value of Wilcoxon’s Statistics**	***p*-Value**
ASF (no of androgenic structures/100 plated anthers)	128.5	0.260
GPR (no of green plants regeneration/100 plated anthers)	154.5	0.040 *
APR (no of albino plants regeneration/100 plated anthers)	126.0	0.265

Signif. codes: 0 ‘**’ 0.01 ‘*’ 0.05.

**Table 3:** The origin and pedigree of the spring and winter wheat genotypes.

Phenotype	Genotype	Source	Pedigree
**Spring**	Tybalt	NSGC	ZE 95-2355/Chablis
	Rescue	NSGC	Apex/S-615
	Fortuna	NSGC	Kenya 58/Newthatch//Frontana/3/Rescue/Chinook
	Leda Collection A47	NSGC	Leda/Hybrid 46
	Ostka Smolicka	PBS	Palermo/KOC 2926/92
	Ruzynska II	NSGC	Unknown-Solid-stemmed-Variety
	Carola	LIPK	Capega/Garant
	AC Abbey	AAFC	BW608/93464//BW591
	Tioga	NSGC	Fortuna/3/ND 4/Rescue//II-50-17/51-3349
	Parabola	MPB	Torka /Henika//Candeza.
	Chinook	NSGC	Thatcher/S615-11
	Glenman	NSGC	Tezanos Pintos Precoz/Sonora 64 (208774C-1R8M)//Fortuna
	Arabella	DPB	Leiffer/Batuta
	Sawtana	NSGC	Rescue//Mida/Cadet
	Sumai 3	NSGC	Funo/Taiwan Xiaomai
**Winter**	Greer	NSGC	WA 4765//Burt/PI 178383
	KS96WGRC36	NSGC	TAM 107/4/TA 870
	Wichita	NSGC	Early Blackhull/Tenmarq
	Geneva	NSGC	Ross wheat (Heine’s VII)/3/(NY5207aB-2B-34) Burt//Genesee/CI 12658/4/Genesee
	Freedom	NSGC	GR-876/OH-217
	Lr19	NSGC	Thatcher*6/Agropyron elongatum
	Augusta	NSGC	B2747 (Genessee/Redcoat) //Yorkstar
	Century	NSGC	Payne//TAM W-101/Amigo
	Antelope	NSGC	Pronghorn/Arlin
	Agrus	NSGC	Trumbull/Agropyron elongatum/4/Fultz sel./3/Trumbull//Hope/Hussar
	Clark	NSGC	Beau//65256A1-8-1/67137B5-16/4/Sullivan/3/Beau//5517B8-5-3-3 /Logan; 65256A1-8-1 = Caldwell sib
	Ozon	DPB	LP-296-4-96/Tambor//Denver
	Hondia	DPB	CHS38337/KOC1284/97
	Karl	NSGC	Plainsman V/3/Kaw/Atlas 50//Parker*5/Agent
	Tam 107	NSGC	TAM 105*4/Amigo

NSGC—National Small Grain Collection, United States Department of Agriculture, Agricultural Research Service Aberdeen-Idaho, USA. LIPK—Leibniz-Institut für Pflanzengenetik und Kulturpflanzenforschung—Gatersleben, Germany. DPB—Danko Plant Breeding Ltd., Poland. MPB—Malopolska Plant Breeding Ltd., Poland. PBS—Plant Breeding Smolice Ltd. IHAR Group, Poland.

## Supplementary Materials

The following are available online at https://www.mdpi.com/2223-7747/9/1/49/s1, Table S1. Average monthly temperatures in the growing season from October 2015 to June 2016 in comparison to average temperatures of 57 years in Poznań Agricultural Research Station, Poland. Table S2. Average monthly rainfall in the growing season from October 2015 to June 2016 in comparison to average rainfall of 57 years in Poznań Agricultural Research Station, Poland. Table S3. A comparison of the efficiency of androgenic structures formation (ASF), green plants regeneration (GPR) and albino plants regeneration (APR) of the influence of growth hormones in the induction media (I and II) on regeneration in the anther cultures of the spring and winter wheat based on right-sided (for Spring genotypes) and left-sided (for Winter genotypes) Wilcoxon test. Table S4. Wilcoxon right-side test comparing efficiency. Figure S1. Androgenic structures (black arrow) with a green plant developing (white arrow) 14 days after passage to regeneration medium (Ac Abbey spring phenotype). Bar represents 1 mm. Figure S2. Androgenic structures (black arrow) with an albino plant developing (white arrow) 14 days after passage to regeneration medium (Ac Abbey spring phenotype). Bar represents 1 mm.
