# Supplementary material for: Comparison of the Androgenic Response of Spring and Winter Wheat (Triticum aestivum L.)
Source: Plants (Basel). 2019 Dec 31;9(1):49. doi: 10.3390/plants9010049 (PMC7020181; doi:10.3390/plants9010049)
Supplement: Supplementary file 1 [file plants-09-00049-s001.pdf]

plants

MDPI

Supplemental Materials

Table S1. Average monthly temperatures in the growing season from October 2015 to June 2016 in comparison to average temperatures of 57 years in Poznań Agricultural Research Station, Poland.

Temperature						
		Decades				
**Year**	**Month**	**I**	**II**	**III**	**Mean**	**Average Temperatures from 57 Years**
2015	October	9,8	6,7	8,1	**8,2**	8,9
	November	6,4	9	2,4	**5,9**	3,8
	December	6,1	5,1	5,7	**5,6**	0,1
2016	January	−6,6	−1,6	2,2	**−2,0**	−1,2
	February	5,5	2,2	2,8	**3,5**	−0,2
	March	2,5	2,5	6,0	**3,7**	3,5
	April	10,2	9,3	6,4	**8,6**	8,8
	May	13,7	12,9	19,2	**15,3**	14,3
	June	18,1	16,4	20,4	**18,3**	17,5

Table S2. Average monthly rainfall in the growing season from October 2015 to June 2016 in comparison to average rainfall of 57 years in Poznań Agricultural Research Station, Poland.

Rainfall						
		Decades				
**Year**	**Month**	**I**	**II**	**III**	**Total**	**Average Rainfall from 57 Years**
2015	October	0	19	0	**19**	37,2
	November	8,7	32,5	12,3	**53,5**	35,8
	December	6,7	15,7	4,7	**27,1**	38,7
2016	January	6,4	16,2	9,0	**31,6**	31,5
	February	6,8	7,6	22,4	**36,8**	27,7
	March	18,6	8,0	22,4	**49,0**	31,7
	April	10,0	24,0	3,4	**37,4**	31,0
	May	16,6	19,4	7,0	**43,0**	50,5
	June	9,6	59,4	14,6	**83,6**	59,4

Table S3. A comparison of the efficiency of androgenic structures formation (ASF), green plants regeneration (GPR) and albino plants regeneration (APR) of the influence of growth hormones in the induction media (I and II) on regeneration in the anther cultures of the spring and winter wheat based on right-sided (for Spring genotypes) and left-sided (for Winter genotypes) Wilcoxon test.

Spring Genotypes - the Right-Sided Wilcoxon Test		
**Parameters**	**The Value of Wilcoxon's Statistics**	***p*-Value**
ASF (no of androgenic structures/100 plated anthers)	140.5	0.127
GPR (no of green plants regeneration/100 plated anthers)	103.5	0.656
APR (no of albino plants regeneration/100 plated anthers)	136.5	0.143
**Winter Genotypes - the Left-Sided Wilcoxon Test**		
**Parameters**	**The value of Wilcoxon's statistics**	***p*-value**
ASF (no of androgenic structures/100 plated anthers)	96.5	0.259
GPR (no of green plants regeneration/100 plated anthers)	94.0	0.207
APR (no of albino plants regeneration/100 plated anthers)	128.0	0.777

Table S4. Wilcoxon right-side test comparing efficiency.

Parameters	Spring Genotypes<br>The Value of Wilcoxon's Statistics	*p*-Value
ASF	18376	0.01024*
GPR	16985	0.1487
APR	18512	< 0.0001***
	**Winter Genotypes**	
ASF	14808	0.9344
GPR	15166	0.9496
APR	16387	0.3245

Signif. codes: 0 '***' 0.001 '**' 0.01 '*' 0.05.

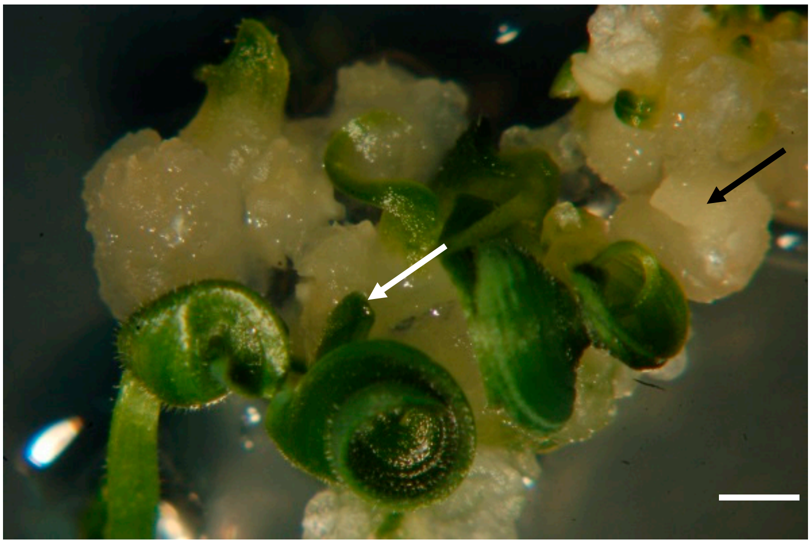

Figure S1. Androgenic structures (black arrow) with a green plant developing (white arrow) 14 days after passage to regeneration medium (Ac Abbey spring phenotype). Bar represents 1 mm.

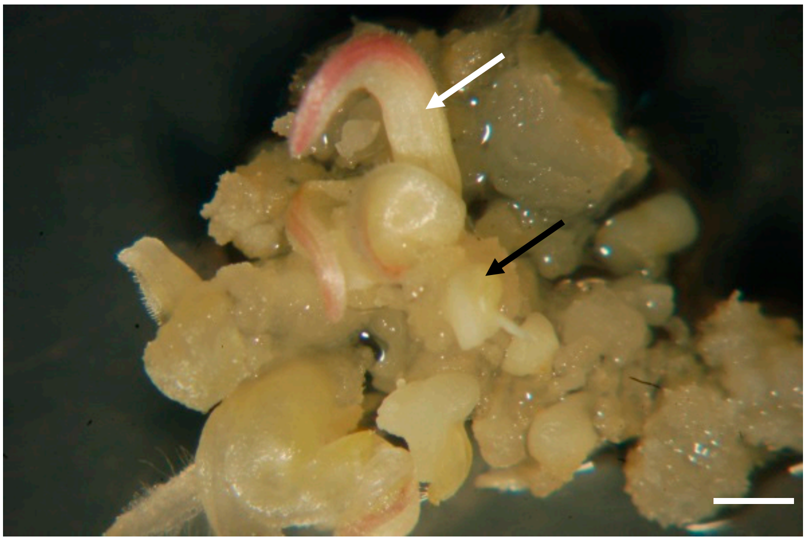

Figure S2. Androgenic structures (black arrow) with an albino plant developing (white arrow) 14 days after passage to regeneration medium (Ac Abbey spring phenotype). Bar represents 1 mm.
